# Extract of *Synedrella nodiflora* (L) Gaertn exhibits antipsychotic properties in murine models of psychosis

**DOI:** 10.1186/s12906-017-1901-2

**Published:** 2017-08-07

**Authors:** Patrick Amoateng, Samuel Adjei, Dorcas Osei-safo, Kennedy K. E. Kukuia, Emelia Oppong Bekoe, Thomas K. Karikari, Samuel B. Kombian

**Affiliations:** 1 Department of Pharmacology and Toxicology, School of Pharmacy, College of Health Sciences, University of Ghana, P.O Box LG 43, Legon, Accra, Ghana; 2 Department of Animal Experimentation, Noguchi Memorial Institute for Medical Research (NMIMR), College of Health Sciences, University of Ghana, P. O. Box LG 581, Legon, Accra, Ghana; 3 Department of Chemistry, School of Physical and Mathematical Sciences, College of Basic and Applied Sciences, University of Ghana, P. O. Box LG 56, Legon, Accra, Ghana; 4 Department of Pharmacognosy and Herbal Medicine, School of Pharmacy, College of Health Sciences, University of Ghana, P.O Box LG 43, Legon, Accra, Ghana; 5 School of Life Sciences, University of Warwick, Coventry, CV4 7AL UK; 6 Midlands Integrative Biosciences Training Partnership, University of Warwick, Coventry, CV4 7AL UK; 7 Department of Pharmacology and Therapeutics, Faculty of Pharmacy, Health Science Center, Kuwait University, Safat, Kuwait

**Keywords:** *Synedrella nodiflora*, chlorpromazine, haloperidol, apomorphine, stereotypy, antipsychotic

## Abstract

**Background:**

The hydro-ethanolic whole plant extract of *Synedrella nodiflora* (SNE) has demonstrated anticonvulsant, sedative and analgesic effects. Preliminary studies conducted in animals, SNE significantly decreased stereotypic behaviours suggesting antipsychotic potential. Coupled with the central nervous system depressant effects of SNE, we hypothesized that it may have utility in the management of psychosis. The present study therefore investigated the antipsychotic potential of the SNE in several murine models of psychosis.

**Method:**

The primary central nervous system activities of SNE (30–3000 mg/kg, p.o) were investigated using the Irwin’s test. The novelty-induced rearing, locomotion and stereotypy counts provoked by SNE (100–1000 mg/kg, p.o) were conducted using the open-field paradigm. The antipsychotic test models used in the screening of SNE (100–1000 mg/kg, p.o) included apomorphine-induced stereotypy, rearing, locomotion and cage climbing activities. The combined effects of a low dose of SNE (100 mg/kg) with various doses of haloperidol and chlorpromazine were analysed using the apomorphine-induced cage climbing and stereotypy, respectively. The ability of SNE to cause catalepsy in naïve mice as well as its effect on haloperidol-induced catalepsy was assessed.

**Results:**

SNE showed acetylcholine-like and serotonin-like activities in the Irwin test, with sedation occurring at high doses. SNE significantly reduced the frequencies of novelty- and apomorphine-induced rearing and locomotion; stereotypy behaviour and the frequency and duration of apomorphine-induced cage climbing in mice. In all the tests performed, SNE was less potent than the reference drugs used (chlorpromazine and haloperidol). In addition, SNE potentiated the effects of haloperidol and chlorpromazine on apomorphine-induced cage climbing and stereotypy activities in mice.

**Conclusion:**

SNE, while exhibiting antipsychotic properties itself, can also potentiate the antipsychotic effects of chlorpromazine and haloperidol.

## Key message

An extract of *Synedrella nodiflora* possesses antipsychotic properties

## Background

Psychosis, a major public health concern, is a chronic recurrent neuropsychiatric disorder that adversely impacts the quality of life of the sufferers [1]. An estimated 2% of people worldwide experience an episode of psychosis in their lifetime, with 80% of these people experiencing the episodes between the ages of 16 and 40 years [2, 3]. Although the aetiology of the disease is unknown, hyperdopaminergic activity is closely linked with the pathogenesis of psychosis [4]. Individuals with psychoses are more prone to suicide, depression, anxiety, aggression, substance abuse, cognitive impairment, victimisation, poverty and increased medical problems [5]. The onset of psychosis is determined by an underlying vulnerability coupled with the impact of environmental stress (including drug/substance abuse), which may trigger active psychotic symptoms, and this provides a basis for the stress/vulnerability model of psychosis [6].

Current antipsychotic drugs only provide symptomatic relief, without altering disease progression [7, 8]. Additionally, the clinical efficacy of these drugs is often limited by adverse reactions such as photo-sensitivity, jaundice, disabling seizures, blindness, agranulocytosis and neuroleptic malignant syndrome [9, 10]. Moreover, the overall functional and quality of life outcomes of patients still remain poor after treatment [5]. Thus, there is a critical need to search for more effective and less toxic therapeutic agents to manage psychosis. An increasing number of herbal products have been introduced into psychiatric practice as alternative or complementary medicines, following the identification of their therapeutic potential and mechanisms of action [11].

*Synedrella nodiflora* (L.) Gaertn (family Asteraceae) is a common shrub found mainly around small rivers and streams as well as along roadsides and under shady trees [12]. In Ghana, traditional medicine practitioners use the aqueous extract obtained after boiling the whole plant for the management of epilepsy and pain. The leaves are also used medicinally to prevent spontaneous abortion, hiccup, as a laxative and feed for livestock [12]. The hydro-ethanolic extract of the whole plant has demonstrated anticonvulsant [13], sedative [14], in vitro antioxidant and free radical scavenging properties [15] as well as anti-nociceptive properties in acute and neuropathic pain [16, 17]. An acute, sub-acute and sub-chronic toxicity of the extract in rodents showed no significant changes in haematological, biochemical and organ histological changes of animals treated with an LD_50_ greater than 6400 mg/kg [18–20]. Our preliminary work conducted on this extract revealed that it was able to reduce stereotypic behaviour characteristic of schizophrenia and other psychotic conditions in experimental animals suggesting that it may have utility in the management of psychotic conditions [21]. Thus, the present study sought to examine, in greater detail, if SNE possessed anti-psychotic potential and if so, how it compared with current clinically available drugs using in vivo models of psychosis in mice.

## Methods

### Plant collection and extraction

Samples of the plant were collected from the Botanical Gardens, University of Ghana, Accra (N5^0^39ʹ32.067 W0^0^11ʹ55.247) in August 2012 and were identified and authenticated at Ghana Herbarium, Department of Botany, University of Ghana, Legon, Accra where a voucher specimen (PA01/UGSOP/GH12) was kept. The hydro-ethanolic extract was prepared as previously described [13, 22]. Briefly, samples of the collected plant were air-dried for 7 days and powdered. Two kilograms of the powder were cold-macerated with 70% *v*/v of ethanol in water. The hydro-ethanolic extract was then evaporated using a rotary evaporator (Buchi Rotavapor® R-300, Flawil, Switzerland) under reduced pressure to remove ethanol. The aqueous portion was frozen at -20 °C and lyophilised (Bench-top Freeze Dryer, Labfreez Instruments Co., Ltd., Beijing, China). A 14% percentage yield of dried extract was obtained, labelled as SNE and kept in a dessicator.

### Qualitative phytochemistry of SNE

The extract (SNE) was screened for the presence of phytochemical constituents such as alkaloids, glycosides, tannins, sterols, flavonoids using determination protocols as previously described [23] and reported [13].

### High performance liquid chromatography (HPLC) of SNE

HPLC analysis was performed on a Perkin Elmer Flexar HPLC, fitted with a PDA detector and a manual injector. The constituents of SNE were separated on a μBondapak C18 Column (150 × 4.6 mm, 3 μm) with mobile phase 0.1% formic acid (A) and Methanol (A). Gradient elution started with 100% A for 10 min and then moved to 50% in 40 min. It was kept at 50% for another 10 min and returned to 100% in 2 min, making a total run time of 62 min. The flow rate was 1 mL/min and the sample injection was 100 uL (0.108 g in 1:4 methanol-H_2_O mixture). The wavelength was set at 315 nm.

### Experimental animals and housing

Female Imprint Control Region (ICR) mice 6–8 weeks old(weight: 20–30 g), were obtained from and maintained at the Department of Animal Experimentation, Noguchi Memorial Institute for Medical Research (NMIMR), University of Ghana, Legon, Accra, where most of the behavioural experiments were performed. The animals were housed in groups of five in stainless steel cages (dimensions: 34 cm × 47 cm × 18 cm) with soft wood shavings as bedding and maintained under laboratory conditions (temperature 22 ± 2 °C, relative humidity 60–70%, and 12 h light-dark cycle). Additionally, the animals were fed with normal commercial pellet diet (AGRIMAT, Kumasi), and given water ad libitum.

In other experiments conducted at the Health Science Center (HSC), Kuwait University, female Balb/c mice (20–30 g) were obtained from and maintained at the Animal Resource Center, HSC, Kuwait University, Kuwait. Similar laboratory conditions as described above were maintained for this set of animals. All experiments were performed during the day between the hours of 8:00–15:00 GMT and complied with internationally recognized and accepted guidelines for the humane handling of experimental animals as contained in those published by the Canadian Council on Animal Care, 1993.

### Chemicals and reagents

The following compounds (source/manufacturer) were used in this study: Chlorpromazine hydrochloride (Renandin, France), haloperidol (STEROP, Belgium) and Apomorphine hydrochloride (Macfarlan Smith Ltd., Scotland, UK).

### Primary observation test

The behavioural and neuroactive effects of SNE were initially evaluated according to standardised observation grid similar to that previously described [24]. Briefly, groups of mice (*n* = 5) were treated with SNE (30, 100, 300, 1000 or 3000 mg/kg, p.o) or vehicle (distilled water, 10 ml/kg, p.o). Observations were performed 15, 30, 60, 120 and 180 min after administration of the test substance and also 24 h later and behavioural modifications, physiologic and neurotoxic symptoms were recorded according to a standardised observation grid derived from Irwin (1968). The grid contained the following parameters: death, convulsions, tremor, Straub tail, sedation, excitation, jumping, abnormal gait (rolling, tiptoe), motor incoordination, altered muscle tone, loss of grasping, akinesia, catalepsy, loss of traction, loss of balance, fore-paw treading, writhing, piloerection, stereotypies (sniffing, chewing, head movements), head-twitches, scratching, altered respiration, aggression, altered fear, altered reactivity to touch, ptosis, exophthalmia, loss of righting reflex, loss of corneal reflex, analgesia, defecation/diarrhoea, salivation, lacrimation, and pupil diameter (myosis/mydriasis).

### Assessment of novelty-induced rearing and locomotor behaviour

The novelty-induced behaviour was evaluated using an open-field observation box (dimensions: 25 cm × 25 cm × 30 cm) made of transparent Perspex and behavioural events recorded using a camcorder and tracked with Behavior Tracker® software. The base of the maze had 16 squares (6.5 cm × 6.5 cm) demarcated with a non-toxic permanent marker.

To conduct this investigation, groups of mice were treated with SNE (100, 300, 1000 mg/kg, p.o) or chlorpromazine 1 mg/kg (i.p) or vehicle (distilled water, 10 ml/kg, p.o). Thirty minutes after the treatments, the animals were placed individually into the open-field observational box and their behaviour recorded for 5 min using a camcorder (Everio™ model, GZ-MG 130 U, JVC, Tokyo, Japan) suspended above the maze with the aid of a stand. Novelty-induced rearing was counted as the number of times the mouse stood on its hind limbs with its forelimbs against the wall of the observation cage (supported rearing) or in free air (unsupported rearing). The number of rearing (both supported and unsupported) was tracked for 5 min. Also, the number of line crossing was counted as a representation of locomotor activity.

### Novelty-induced stereotypy

The number and total duration of stereotypic behaviour exhibited by the mice pre-treated with SNE (10, 100 or 1000 mg/kg, p.o) or vehicle (distilled water, 10 ml/kg, p.o) over two-hour periods was assessed in an automated open-field test (VersaMax Animal Activity Monitoring System, AccuScan Instrument Inc., USA) as previously reported [14]. This test system comprised four animal monitoring chambers (16 in × 16 in × 12 in) covered by transparent lids with perforations, an analyser and a computer. The base of each monitoring chamber was lined with vertical and horizontal laser generators and sensors. The behaviours of interest were pre-configured into the system. During the test, any behaviour exhibited by the test animals through beam interruptions were transmitted to the analyser, recorded on a computer, and the data subsequently exported to Microsoft Excel. Our experimental setup enabled the researcher to test the same animal under three different experimental conditions (primary, secondary and auxillary in succession). In the experiments, primary and secondary sessions were conducted for 60 and 120 min respectively. The test animals were made to acclimatise by undergoing a two-day procedure in the system without drug administration. On the third day, after an initial 60 min primary session, the mice were treated with SNE or distilled water (as a control) and tested for a 120 min secondary session. When the animal broke the same beam (or set of beams) repeatedly then the monitor considered the animal as exhibiting stereotypy. This typically happened during grooming, head bobbing, sniffing, gnawing, etc.

### Apomorphine-induced locomotor and rearing activity

Using the experimental set-up as described in the novelty-induced locomotor and rearing activities, mice were pre-treated with SNE (100, 300 or 1000 mg/kg, *p.o*) or chlorpromazine (0.1, 0.3, or 1.0 mg/kg, i.p) or vehicle (distilled water, 10 ml/kg, *p.o*) and 30 min later, they received apomorphine (2 mg/kg, i.p) and placed in the open field test chamber. A vehicle group without apomorphine administration was also included. The events were recorded with a camcorder for 30 min and the videos tracked for the frequency of rearing and line-crossings.

### Apomorphine–induced stereotypy

This procedure was performed as previously described [25]. Briefly, mice were pre-treated with various doses of the extracts (10, 100, 1000 mg/kg, p.o), chlorpromazine (0.1, 0.3, and 1 mg/kg, i.p) or vehicle (10 ml/kg, p.o). Thirty minutes later, apomorphine (2 mg/kg, i.p) was administered to each mouse and placed into an observation cage and events recorded for 30 min. The frequency of stereotypic behaviour, mainly sniffing and gnawing, were tracked and scored. The stereotypic episodes were scored as follows: absence of stereotypy (0), sniffing (1), gnawing (2) and staying on the same spot (3). The frequency of these behaviours were measured and weighted as a product of the frequency and the behaviour score. A total of all the scores were made for each mouse and a mean of 5 min period calculated and plotted as described [26]. An antagonism of the classic stereotypic behaviours of a low dose of apomorphine indicated neuroleptic (antipsychotic) activity.

In a separate experiment, combination of a dose of SNE lower than its anti-psychotic ED_50_ dose (i.e SNE 100 mg/kg, p.o) with three doses of chlorpromazine (0.1, 0.3 and 1.0 mg/kg, i.p) were evaluated using the fixed ratio method [27] for synergism, addition or antagonism. The effect of the combinations of SNE and chlorpromazine was assessed for antipsychotic effects using the apomorphine-induced stereotypy test as described above.

### Apomorphine-induced cage climbing

This method as described previously [28] was employed with minor modifications. Briefly, mice were treated with SNE (100, 300, 1000 mg/kg, p.o), haloperidol (0.1, 0.3, 1 mg/kg, i.p) or vehicle (distilled water, 10 ml/kg, p.o) and injected with apomorphine (2 mg/kg, i.p) 30 min later and immediately placed individually into an all wire-meshed cage (mesh size: 1 cm × 1 cm; dimensions = 27 cm × 20 cm × 20 cm). A camcorder placed above the cage was used to record animal behaviour in the cage for 30 min post-apomorphine injection and the video recording tracked the frequency and duration of climbing. In a preliminary study done in our laboratory, haloperidol was found to be more potent than chlorpromazine in reducing the frequency and duration of cage climbing, thus in this assay haloperidol was used as the reference antipsychotic drug.

In a separate experiment using the above experimental protocol, a combination of low dose SNE (100 mg/kg, p.o) and three doses of haloperidol (0.1, 0.3 and 1.0 mg/kg, i.p) were evaluated for synergism, addition or antagonism using the fixed ratio method [27].

### Extract/drug-induced catalepsy

The ability of SNE to induce catalepsy in mice was evaluated as previously described [29, 30]. Briefly, mice treated with the SNE (100, 300, 1000 mg/kg, p.o), haloperidol (0.1, 0.3 1.0 mg/kg, i.p) or vehicle were tested individually on the catalepsy set-up made of a Perspex rod elevated over a height of 3.5 cm. 30 min posttreatment, the mice were placed individually on the rod with their fore-paws and time spent by each mouse in that position recorded until the animal removed its fore-paws from the rod unto the floor or climbed the rod, indicating the end of catalepsy. This procedure was repeated at 30, 60 and 120 min post-treatment.

### Haloperidol-induced catalepsy

The effect of SNE on haloperidol-induced catalepsy was performed as previously described with a slight modification [30, 31]. In brief, mice were pre-treated with SNE (100, 300 or 1000 mg/kg, p.o) or vehicle (distilled water, 0.01 ml/kg, p.o) and 30 min later each mouse was treated with haloperidol 1 mg/kg (i.p) and tested for catalepsy as described above.

## Statistical analysis

The ED_50_ (concentration responsible for 50% of the maximal effect) of extract/reference drug was determined using an iterative computer least squares method in Prism for Windows version 5.0 (GraphPad Software, San Diego, CA, USA) with the following nonlinear regression (four-parameter logistic equation).

$$ Y=\frac{a+\left(a-b\right)}{1+{10}^{\left(\left( Log\;{ED}_{50}-X\right)\times Hill\kern0.5em  Slope\right)\Big)}} $$

Where, X is the logarithm of concentration. Y is the response, starting at ***a*** and ending at point ***b*** with a sigmoid shape.

The fitted midpoints (ED_50_s) of the curves were compared statistically using the *F* test.

Statistical analyses (one- or two-way ANOVA followed by an appropriate post hoc test) were conducted using Prism 5.0, with *P* ≤ 0.05 considered statistically significant. Graphs were plotted using Sigma Plot for Windows version 11.0 (Systat Software Inc., Germany).

## Results

### Qualitative phytochemistry of SNE

SNE was found to contain flavonoids, tannins, saponins, alkaloids, cardiac glycosides, coumarins, triterpenes, sterols, anthraquinones and phenolic compounds as previously reported [13].

### High performance liquid chromatography (HPLC) of SNE

**Fig. 1:**
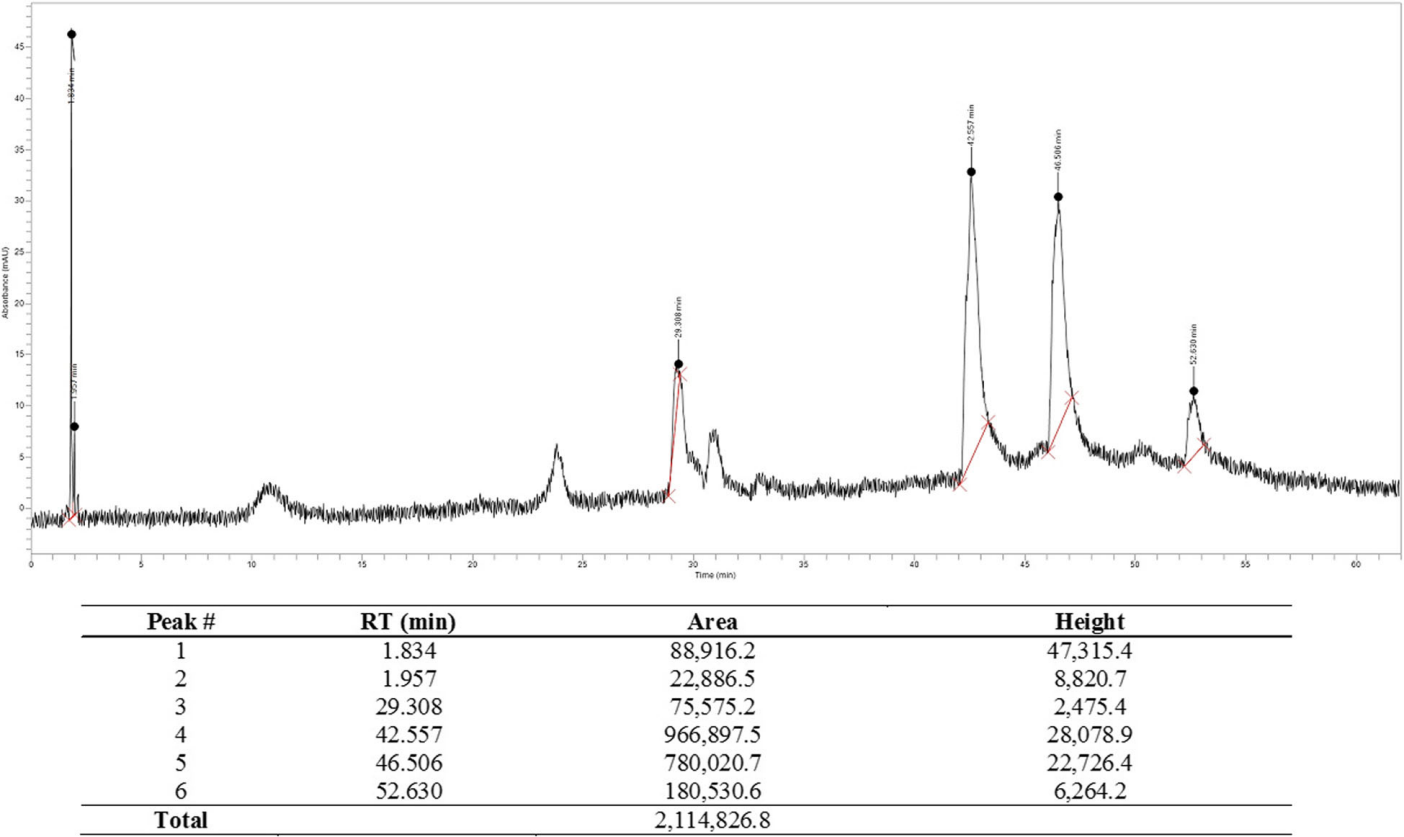
HPLC chromatogram of SNE monitored at 315 nm

Figure 1 shows the HPLC chromatogram of SNE. Two major constituents were observed at retention times 42.56 and 46.51 min. The percentage composition of these constituents was determined to be 45.72% and 36.88% respectively, using the areas under the curve (AUCs).

### Primary observation test

SNE-treated mice primarily showed defaecation and fore-paw treading at all doses 30–3000 mg/kg with sedation. There were no convulsion, ptosis, motor incoordination, stereotypic behaviour or death in the animals pre-treated with the extract at the 0–180 min and 24 h observational period.

### Assessment of novelty-induced rearing and locomotor behaviour

There was a significant decrease in the frequencies of rearing (*P* < 0.0001, F_4,16_ = 25.83, Fig. 1) and line-crossing (*P* = 0.0001, F_3,15_ = 14.62, Fig. 1) behaviours in the SNE-treated and chlorpromazine (1 mg/kg)-treated mice in comparison with the vehicle-treated group. The percentage decrease in the frequencies of rearing and line-crossing for SNE (100, 300 and 1000 mg/kg) and CPZ (1 mg/kg) were 0%, 32%, 3% and 77% respectively [rearing] and 42%, 50%, 36% and 70% respectively [line-crossing]. The decreased effects in the SNE-treated mice were not dose-dependent (Fig. 1).

### Apomorphine-induced locomotor and rearing activity

**Fig. 2:**
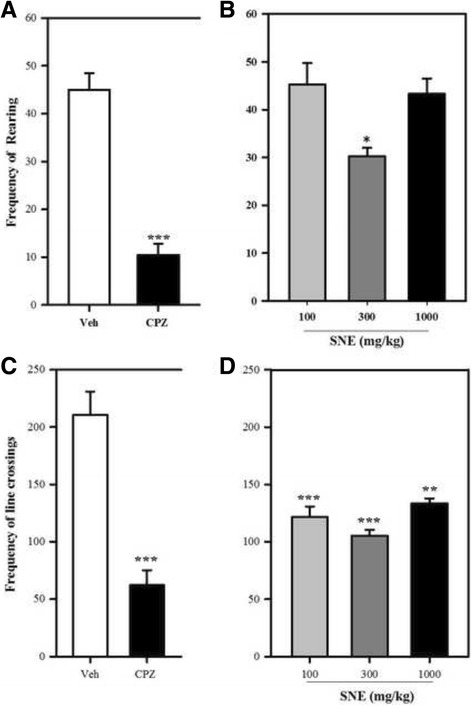
Frequency of rearing (**A** and **B**) and line-crossings (**C** and **D**) of mice treated with chlorpromazine (CPZ; 1 mg/kg, i.p), SNE and vehicle (Veh) when observed for 5 min in an open-field paradigm. Data are mean ± SEM (*n* = 5). *P ≤ 0.05, ** *P* ≤ 0.01, ***P ≤ 0.001 compared with vehicle group (one-way ANOVA followed by a Dunnett’s multiple comparison post hoc test)

Apomorphine induced a characteristic increase in rearing and locomotor activity in the treated group when compared with mice treated with the vehicle (i.e no apomorphine) (Fig. 2a and d). Pretreatment of mice with SNE resulted in a significant reduction in the frequency of apomorphine-induced rearing behaviour (*P* = 0.0011, F_3,15_ = 9.103, Fig. 2b) and line-crossing (an indicator of locomotor activity; *P* = 0.0013, F_3,12_ = 10.12, Fig. 2e). The percentage of reductions in rearing activity by SNE (100, 300, 1000 mg/kg) were 68%, 58% and 61% respectively and those for line-crossing were 60%, 70% and 49% respectively. Likewise, chlorpromazine significantly reduced apomorphine-induced rearing (*P* = 0.0065, F_3,15_ = 6.067, Fig. 2c) and locomotor activity (*P* = 0.007, F_3,12_ = 6.58, Fig. 2f) in mice that were pretreated with it. The percentage of reductions in rearing activity by CPZ (0.1, 0.3, 1.0 mg/kg) were 31%, 50% and 75% respectively and those for line-crossing were 35%, 42% and 28% respectively.

### Novelty-induced stereotypy

**Fig. 3:**
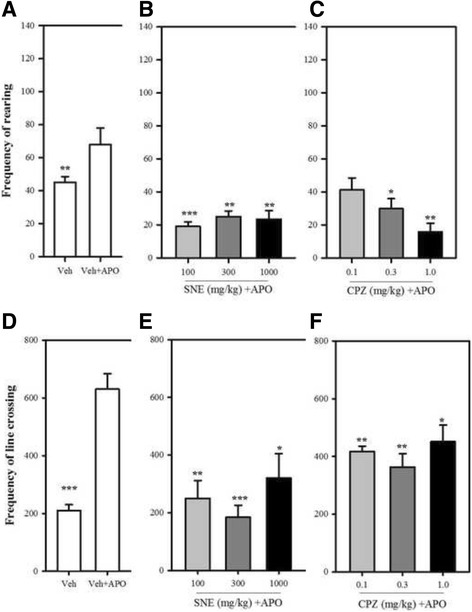
Effect of SNE, CPZ and vehicle (Veh) (distilled water, 0.01 ml/kg, p.o) on the frequency of rearing (**A, B** and **C**) and line-crossings (**D, E** and **F**) of mice, 30 min post apomorphine (APO) (2 mg/kg, i.p) treatment in an open-field paradigm. Data are mean ± SEM (*n* = 5). *P ≤ 0.05, ** *P* ≤ 0.01, ***P ≤ 0.001 compared with Veh + APO group (one-way ANOVA followed by a Dunnett’s multiple comparison post hoc test)

The frequency of stereotypy in the mice prior to being treated with SNE or vehicle were not significantly different from each other for the 60 min primary (no-drug) session (Fig. 3?). However, 10 min after the SNE administration, there was a significant drop (77%; *P* < 0.0001, F_3,68_ = 11.02, Fig. 3a) in the frequency of stereotypy in the mice treated with SNE 1000 mg/kg. This drop was 92% at 20 min post SNEand was sustained throughout the 120 min observation period. However, at the 100 mg/kg dose level 10 min post administration, the decrease in stereotypy count was 84% and lasted for only 60 min before returning nearly to the baseline by the 180th min. Finally, for the 10 mg/kg SNE-treated mice, stereotypy count declined by 56% at 30 min and recovered to baseline. The overall effect, as calculated from the AUC from the time-course curves for each dose of SNE, indicated a significant dose-dependent decrease in the stereotypy count (*P* = 0.0002, F_3,16_ = 12.10, Fig. 2b). A similar trend was observed when the total duration of stereotypy was measured (Fig. 2c & d).

### Apomorphine–induced stereotypy

**Fig. 4:**
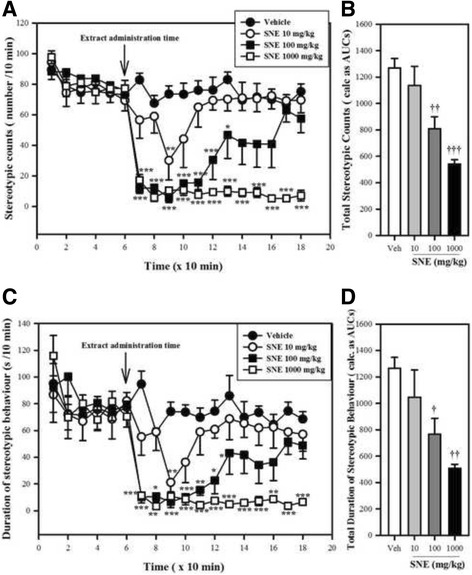
Effects of SNE on the frequency (**A**) and duration(**C**) of stereotypy in mice. Graphs **A** and **C** are the time-course effects of varying doses of SNE recorded over 180 min. Graphs **B** and** D** indicate the total frequency and duration of stereotypy behaviour (calculated as AUCs from the time-course graphs). Data are mean ± SEM (*n* = 5). *P ≤ 0.05, ** *P* ≤ 0.01, ***P ≤ 0.001 compared with vehicle group (Two-way ANOVA followed by a Bonferroni’s *posthoc* test). ^†^ *P* ≤ 0.05, ^††^ *P* ≤ 0.01, ^†††^ *P* ≤ 0.001 compared with vehicle group (one-way ANOVA followed by a Dunnett’s multiple comparison post hoc test)

**Fig. 5:**
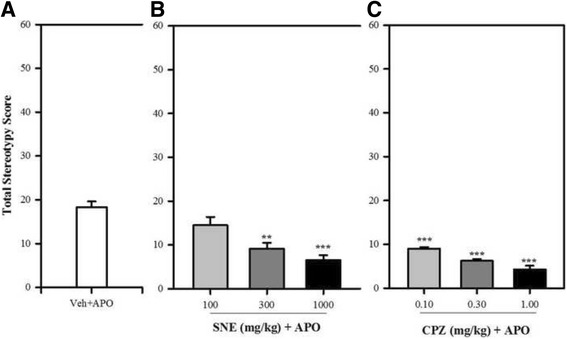
Effect of SNE, CPZ and vehicle (Veh) (distilled water, 0.01 ml/kg, p.o) on the stereotypy score of mice pre-treated (30 min) with apomorphine (APO) (2 mg/kg, i.p). Data are mean ± SEM (*n* = 5). *P ≤ 0.05, ** *P* ≤ 0.01, ***P ≤ 0.001 compared with Veh + APO group (one-way ANOVA followed by a Dunnett’s multiple comparison post hoc test)

Apomorphine-induced stereotypic behaviours in mice was characterised by sniffing and gnawing. SNE and CPZ significantly decreased the apomorphine-induced stereotypy scores in the treated mice (SNE: *P* = 0.0007, F_3,14_ = 10.64, Fig. 4b; CPZ: *P* < 0.0001, F_3,16_ = 51.19, Fig. 4c). The percentage decrease in the stereotypy scores by SNE 100, 300 and 1000 mg/kg were 20%, 49% and 64% respectively; and that for CPZ 0.1, 0.3 and 1.0 mg/kg were 50%, 66% and 76%, respectively. SNE was less potent than CPZ in this model (Fig. 5).

**Fig. 6:**
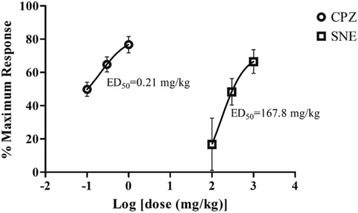
A dose-response effect of SNE (100–1000 mg/kg) and CPZ (0.1–1 mg/kg) against apomorphine-induced stereotypy behaviour in mice. Data are mean ± SEM (*n* = 5)

Interestingly, the antipsychotic effect of CPZ in combination with SNE 100 mg/kg was superior to that of CPZ alone (i.e 1.3 times more potent) (Fig. 6).

### Apomorphine-induced cage climbing

**Fig. 7:**
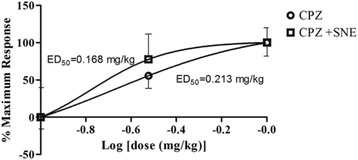
A dose-response effect of CPZ (0.1–1 mg/kg) alone and CPZ (0.1–1 mg/kg) + SNE 100 mg/kg against apomorphine-induced stereotypy behaviour in mice. Data are mean ± SEM (*n* = 5)

Pretreatment with SNE (100, 300 and 1000 mg/kg) significantly and dose-dependently reduced the total frequency of climbing (*P* < 0.0001, F_3,16_ = 37.10; Fig. 7b) by 58%, 80% and 94% respectively. SNE (100, 300 and 1000 mg/kg) also significantly and dose-dependently reduced the duration (*P* < 0.0001, F_3,13_ = 30.31; Fig. 7e) of cage climbing activities in the treated mice by 7%, 29% and 88% respectively. Haloperidol (HAL) also exhibited similar effects on frequency of cage-climbing (*P* < 0.0001, F_3,16_ = 172.10, Fig. 7c) with an 84%, 98% and 100% decrease for the three dose levels respectively; and duration of cage-climbing (*P* < 0.0001, F_3,15_ = 146.70, Fig. 7f) with an 26%, 100% and 100% reduction for the three dose levels respectively. ED_50_ values of the frequency and duration of cage-climbing showed that SNE was less potent in comparison to HAL (Table 1) in this model.

**Table 1:** ED_50_ values (mg/kg) ± SEM of SNE, haloperidol (HAL) and haloperidol (HAL) (0.1, 0.3, 1.0 mg/kg, i.p) + SNE (100 mg/kg, p.o) [HAL + SNE] on the frequency and total duration of apomorphine-induced cage climbing in mice

	SNE	HAL	HAL+ SNE
Frequency	170.0 ± 1.50	0.1391 ± 1.07	0.0969 ± 1.08
Duration	349.1 ± 0.03	0.1047 ± 1.61	0.1439 ± 1.70

**Fig. 8:**
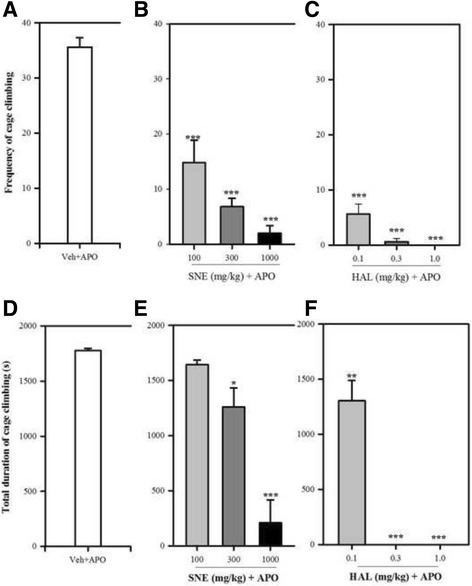
Effect of SNE, HAL and vehicle (Veh) on the total frequency and duration of cage climbing of mice, 30 min post apomorphine (APO) (2 mg/kg, i.p) treatment. Data are mean ± SEM (*n* = 5). *P ≤ 0.05, **P ≤ 0.01, ***P ≤ 0.001 compared with Veh + APO group (one-way ANOVA followed by a Dunnett’s multiple comparison post hoc test)

**Fig. 9:**
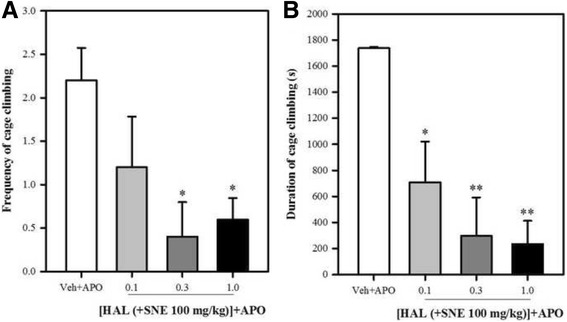
Effect of haloperidol (HAL) when combined with SNE and vehicle on the total frequency (**A**) and duration (**B**) of cage climbing of mice, 30 min post apomorphine (APO) (2 mg/kg, i.p) treatment. Data are mean ± SEM (*n* = 5). *P ≤ 0.05, **P ≤ 0.001 compared with vehicle group (one-way ANOVA followed by a Dunnett’s multiple comparison post hoc test)

There was a significant reduction in the total frequency [% reductions are 96%, 98% and 98% for 0.1, 0.3 and 1.0 mg/kg HAL respectively] and duration [% reductions are 60%, 83% and 86% for 0.1, 0.3 and 1.0 mg/kg HAL respectively] of cage climbing in the mice that received HAL (0.1, 0.3 or 1.0 mg/kg) + SNE(100 mg/kg) [(frequency: *P* = 0.033, F_3,16_ = 3.733; Fig. 8a), (duration: *P* = 0.003, F_3,15_ = 7.136; Fig. 8b)]. The ED_50_ (mg/kg) values for the frequency and duration of cage climbing activities of haloperidol (see Fig. 9) was decreased and increased respectively when SNE 100 mg/kg (p.o) was administered with haloperidol in comparison to haloperidol alone (Table 1).

### Catalepsy model

Haloperidol (0.1, 0.3 and 1 mg/kg) exhibited a significant and dose-dependent cataleptic behaviour in the treated mice (*P* = 0.01, Table 2). One-way ANOVA analysis showed that SNE (100 and 1000 mg/kg) produced a non-significant cataleptic behaviour. However, at 300 mg/kg, SNE by the 60th min produced a significant (two-way ANOVA analysis, *P* ≤ 0.01) cataleptic effect. (Table 2).

**Table 2:** Effect of extracts SNE, haloperidol (HAL) and vehicle on the duration of catalepsy

Treatment (mg/kg)	Time (s)			
	15th min	30th min	60th min	
Vehicle	0.000 ± 0.00	0.00 ± 0.00	0.00 ± 0.00	-
SNE 100	0.000 ± 0.00	2.038 ± 2.04	2.448 ± 1.05	
SNE 300	0.000 ± 0.00	2.146 ± 2.15	5.862 ± 2.95**	
SNE 1000	0.000 ± 0.00	0.000 ± 0.00	1.690 ± 1.69	
HAL 0.3	34.187 ± 8.01	87.902 ± 33.81	108.892 ± 32.84	
HAL 1.0	154.340 ± 42.16**	153.273 ± 52.08*	125.403 ± 55.14*	
HAL 3.0	162.660 ± 46.41**	179.510 ± 50.24**	192.122 ± 40.87**	

Data are mean ± SEM (*n* = 5). *P ≤ 0.05, ** *P* ≤ 0.01, compared with vehicle group (two-way ANOVA followed by a Bonferroni’s *posthoc* test)

### Haloperidol-induced catalepsy

**Fig. 10:**
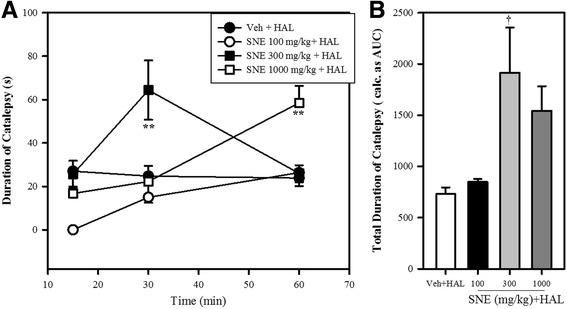
Effects of SNE on haloperidol (HAL)-induced catalepsy in mice. Left paneled graph (A) is the time-course effects 15, 30 and 60 min post haloperidol administration. Column graph (B) is the total duration of catalepsy (calculated as AUCs from the time-course graphs). Data are mean ± SEM (*n* = 5). ** *P* ≤ 0.01 compared with vehicle group (two-way ANOVA followed by Bonferroni *posthoc* test). ^†^ *P* ≤ 0.05 compared with vehicle group (one-way ANOVA followed by a Dunnett’s multiple comparison post hoc test)

Administration of SNE (100 mg/kg), 15 min after the administration of HAL 1.0 mg/kg, completely inhibited (100%) the cataleptic effect of HAL as exhibited in the Veh + HAL group of mice. At 30 min post HAL administration, this inhibition had dropped to 39% and by the 60th min, only 9% cataleptic activity had been recorded. However, a two-way ANOVA failed to produce any significance when these events produced by SNE 100 mg/kg on HAL were compared to that produced by Veh + HAL. SNE 300 mg/kg also inhibited the cataleptic effect of HAL by 4% at 15 min [not significant, *P* > 0.05] but caused a significant 61% increase in the cataleptic behaviour at 30 min (*P* ≤ 0.01, two-way ANOVA followed by a Bonferroni’s post hoc test; Fig. 10a). However, by the 60th min, only an 8% increase in cataleptic behaviour was recorded. SNE 1000 mg/kg also caused an inhibition of cataleptic behaviour by 37% and 10% at 15 and 30 min post HAL treatment respectively. However, SNE 1000 mg/kg significantly increased the cataleptic event at the 60th min (*P* ≤ 0.01, two-way ANOVA followed by a Bonferroni’s post hoc test; Fig. 10a). The overall assessment of effect of SNE on HAL-induced cataleptic behaviour (i.e total duration of catalepsy calculated as AUCs) suggested that cataleptic behaviour of HAL was increased by SNE 100, 300 and 1000 mg/kg by 13%, 61% and 52% respectively (*P* = 0.029, F_3,8_ = 5.11, Fig. 10b) and this increase was significant (*P* < 0.05) only for SNE 300 mg/kg.

## Discussion

The findings reported in this study provide initial evidence demonstrating that SNE has antipsychotic potential when tested in murine models of schizophrenia. SNE also potentiated the antipsychotic activities of reference antipsychotic drugs; haloperidol and chlorpromazine, suggesting it may enhance the actions of current antipsychotic agents when used in combination. On the other hand, such combinations may also exacerbate the side/adverse effects associated with the use of these agents.

The Irwin test primarily evaluates the qualitative effects of a test substance on the behaviour and physiological functions of experimental animals, ranging from the first doses that have observable effects up to doses that induce clear behavioural toxicity or even death [24]. The test also permits a reasonable estimate of the test substance’s duration of action on the different outcome parameters [32]. In this study, the extract induced defaecation and fore-paw treading at doses 30–3000 mg/kg suggesting cholinergic and serotonergic mechanisms, respectively [33, 34]. The sedation observed at a high dose of 3000 mg/kg also suggests GABAergic or serotonergic mechanisms [35]. Earlier reports of the neuropharmacological properties of the extract confirmed the sedative effect observed here [14].

Rearing behaviour in mice is regulated by multiple neurotransmitter systems including GABAergic (GABA_A_), opioidergic and dopaminergic (D_2_) systems and receptors [36]. Locomotor activity in experimental animals are generally reduced by central nervous system (CNS) depressants and increased by CNS stimulants [37, 38]. Drug-induced decrease in locomotion of experimental animals may be due to decreased motor effects and/or increased sedation [8]. We are not certain which of these two possibilities account for SNE’s effect.

Stereotypy behaviour is one of the key features of psychosis and in humans, it manifests as repetitive performance of strange gestures such as asking the same questions or making the same kind of comments [8]. The ability of SNE to reduce stereotypy count and duration is the first indication of its possible antipsychotic potential.

Apomorphine, a non-selective dopamine receptor agonist, induces changes in animal behaviour and is a test model that provides predictive validity for antipsychotic drug screening [39–42]. Administration of apomorphine in mice can produce increased locomotor activity [43], stereotyped behaviours [44], rearing/grooming [45] and cage-climbing behaviours [46, 47]. In the present study, apomorphine was used as the agent for inducing psychotic/schizophrenic-like behaviours in mice. Psychosis has been linked with increased dopaminergic and serotonergic neurotransmissions, and both preclinical and clinical investigations have confirmed their role in the development of the disease [7, 48]. Apomorphine directly activates post-synaptic dopamine D_2_ receptors in the brain, and by this mechanism low dose of apomorphine (2 mg/kg, s.c) increases locomotor activity and produce stereotypy behaviour, resulting in restricted and persevering behavioural pattern [49]. SNE blunted or reduced the apomorphine-induced rearing, locomotion, stereotypy and cage climbing suggesting that it may have antipsychotic/antischizophrenic activity. Since apomorphine acts by activating dopamine receptors, it is plausible that SNE works as a dopamine receptor antagonist. However, this hypothesis would need to be tested using appropriate models e.g. receptor-binding assays.

The catalepsy test has been used to predict major tranquillizer activity as well as to evaluate motor effects of drugs, particularly related to the extra-pyramidal system [29]. Catalepsy is one of the major adverse effects associated with the use of conventional antipsychotic drugs [50]. Thus, testing for cataleptic behaviour forms an integral part of the discovery and development of antipsychotic drugs. The apparent lack of cataleptic effect of SNE at 100 mg/kg in both naïve and haloperidol-treated mice suggests that the extract at lower doses may not produce any significant motor side-effects. This dose was also able to reduce haloperidol-induced catalepsy. The significant appearance of cataleptic event in naïve mice at 60 min post-SNE 300 mg/kg administration and its significant increase in haloperidol-induced catalepsy also at the 60th min may suggest that motor side effects are likely to develop with higher doses of the extract [31]. Serotonergic, cholinergic and dopaminergic (D_2_ receptor antagonism in the nigrostriatal dopaminergic pathway) systems have been implicated as mechanisms in drug-induced catalepsy or drug-enhanced haloperidol-induced catalepsy [51–54]. SNE’s ability to cause catalepsy does not place it in the “typical” antipsychotic category since this major side effect is shared by both “typical” and most “atypical” antipsychotic agents such as risperidone, aripiprazole, olanzapine, amisulpride, ziprasidone and sertindole [55–59]. Several clinical studies have actually indicated a less significant difference between first generation/typical and second generation/atypical antipsychotic drugs in the incidences of extrapyramidal side effects (EPS). Thus, the use of whether an antipsychotic agent induces EPS or not as the basis of classification needs re-examination [60–64]. Since the effects demonstrated here are from an extract, future isolation of active constituents of this extract responsible for the cataleptic activity would provide a much clearer understanding of SNE’s mechanism of action to produce catalepsy, and hence its classification as either “typical” or “atypical”. The fact that the cataleptic effect of SNE is dose-dependent and hence absent at a low dose of 100 mg/kg of the extract is also worth mentioning.

The combination of CPZ and SNE showed potentiation of CPZ’s antipsychotic-like activities in the tested models as the ED_50_ value of CPZ alone was significantly higher than the CPZ–SNE combination. Thus, SNE can potentially be used by patients on CPZ enabling a reduction in CPZ dose. Furthermore, SNE had a similar effect on the ED_50_ of HAL when combined with it. This also suggests SNE’s effects are not specific to the agent but possibly on the underlying pathophysiology, again suggesting that the extract may be used to reduce the requirement of these clinically established antipsychotic drugs in clinical settings. However, since SNE causes catalepsy (at high doses) just as much as HAL and CPZ, the doses of these orthodox drugs must be reduced if combined with SNE to prevent exaggerated extrapyramidal motor side effects while possibly enhancing the therapeutic outcome. Further clinical studies are required to determine if such combination produce a superior clinical outcomes (e.g. better control of symptoms or less side effects) than conventional therapies alone.

## Conclusion

The hydro-ethanolic extract of *Synedrella nodiflora* whole plant possesses antipsychotic properties. This finding necessitates further investigations into the therapeutic potential and elucidation of mechanisms of action of the extract. Furthermore, chemical isolation, purification and identification of its active phyto-constituents may lead to the discovery of new molecular entities for future development as antipsychotic agents.

## Abbreviations

CPZ: chlorpromazine
HAL: haloperidol
SNE: *Synedrella nodiflora* extract
VEH: vehicle
